# Role of 3-Hydroxy Fatty Acid-Induced Hepatic Lipotoxicity in Acute Fatty Liver of Pregnancy

**DOI:** 10.3390/ijms19010322

**Published:** 2018-01-22

**Authors:** Sathish Kumar Natarajan, Jamal A. Ibdah

**Affiliations:** 1 Department of Nutrition and Health Sciences, University of Nebraska-Lincoln, Lincoln, NE 68583-0806, USA; snatarajan2@unl.edu; 2 Division of Gastroenterology and Hepatology, University of Missouri, Columbia, MO 65212, USA; 3 Department of Medical Pharmacology and Physiology, University of Missouri, Columbia, MO 65212, USA; 4 Harry S. Truman Memorial Veterans Medical Center, Columbia, MO 65201, USA

**Keywords:** acute fatty liver of pregnancy, 3-hydroxy fatty acids, lipoapoptosis, fatty acid oxidation

## Abstract

Acute fatty liver of pregnancy (AFLP), a catastrophic illness for both the mother and the unborn offspring, develops in the last trimester of pregnancy with significant maternal and perinatal mortality. AFLP is also recognized as an obstetric and medical emergency. Maternal AFLP is highly associated with a fetal homozygous mutation (1528G>C) in the gene that encodes for mitochondrial long-chain hydroxy acyl-CoA dehydrogenase (LCHAD). The mutation in LCHAD results in the accumulation of 3-hydroxy fatty acids, such as 3-hydroxy myristic acid, 3-hydroxy palmitic acid and 3-hydroxy dicarboxylic acid in the placenta, which are then shunted to the maternal circulation leading to the development of acute liver injury observed in patients with AFLP. In this review, we will discuss the mechanistic role of increased 3-hydroxy fatty acid in causing lipotoxicity to the liver and in inducing oxidative stress, mitochondrial dysfunction and hepatocyte lipoapoptosis. Further, we also review the role of 3-hydroxy fatty acids in causing placental damage, pancreatic islet β-cell glucolipotoxicity, brain damage, and retinal epithelial cells lipoapoptosis in patients with LCHAD deficiency.

## 1. Fatty Acid Oxidation

Humans have three major types of fatty acid oxidation pathways that feeds high energy reducing equivalents to the mitochondria for the generation of ATP. Mitochondrial β-oxidation, peroxisome β-oxidation, and microsomal ω-oxidation pathways are the three types of oxidation for long chain fatty acids.

### 1.1. Mitochondrial Fatty Acid Oxidation

**Figure 1:**
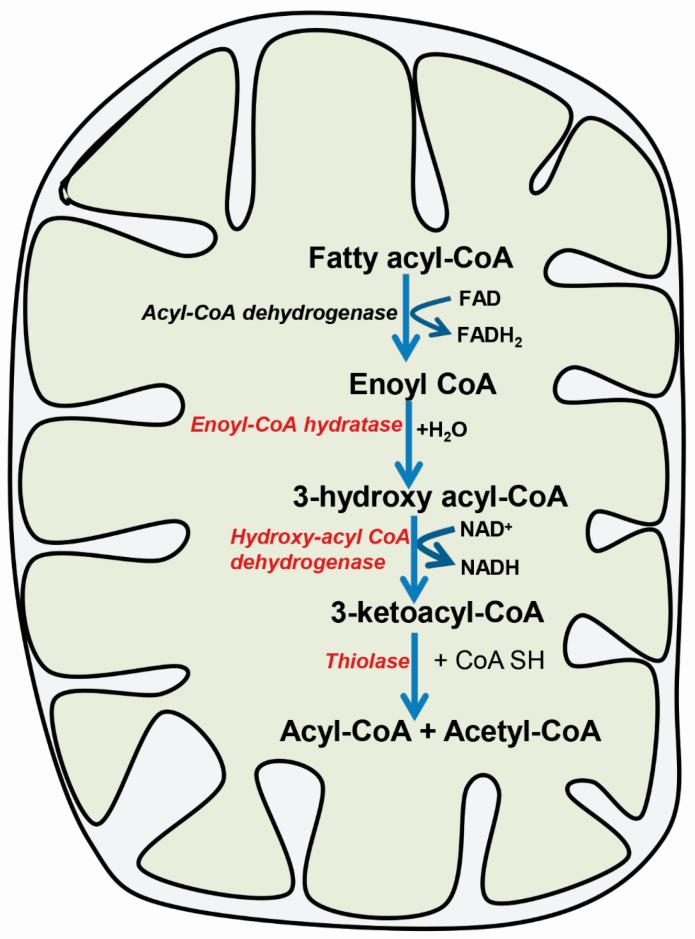
Mitochondrial fatty acid β-oxidation pathway. Classical β-oxidation pathway involves dehydrogenase by acyl-CoA dehydrogenase and hydration, dehydrogenation and thiolyic cleavage is catalyzed by the mitochondrial trifunctional protein (MTP, highlighted in red color). MTP consists of enoyl-CoA hydratase, hydroxy acyl-CoA dehydrogenase and thiolase activity. The straight arrows represent products and bent arrows represent the involvement of co-factor in this enzyme catalyzed reaction.

Mitochondrial long chain fatty acid β-oxidation is the predominant cellular oxidation pathway with the production of acetyl-Coenzyme A (CoA), which feeds into the tricarboxylic acid (TCA) cycle for the high-energy ATP production via the mitochondrial electron transport chain. Fatty acids are acylated and activated by fatty acyl-CoA synthetase in the outer mitochondrial membrane. Carnitine acyl transferase I converts acyl-CoA into fatty acyl carnitine, which is translocated across the inner mitochondrial membrane by carnitine translocase. Carnitine acyl transferase II in the inner mitochondrial membrane catalyzes the formation of fatty acyl-CoA for the initiation of the β-oxidation pathway [1,2,3,4]. Classic mitochondrial fatty acid β-oxidation involves a four-step process: dehydrogenation, hydration, dehydrogenation and thiolytic cleavage (Figure 1). Acyl-CoA dehydrogenase initiates β-oxidation by catalyzing the first dehydrogenation step, forms a double bond, and converts fatty acyl-CoA into trans-enoyl-CoA. The next three steps in the β-oxidation of long chain fatty acids are catalyzed by mitochondrial tri-functional protein (MTP). MTP is a complex heterooctamer protein attached to the inner mitochondrial membrane with 4α-subunits and 4β-subunits encoded by the *HADHA* and *HADHB* gene, respectively. The α-subunit contains long chain enoyl-CoA hydratase activity in its amino-terminal domain, while long chain hydroxy acyl-CoA dehydrogenase (LCHAD) activity resides at the carboxyl-terminal domain. The β-subunit contains the long chain 2-ketoacyl-CoA thiolase activity, which catalyzes the fourth step in β-oxidation cycle. Enoyl-CoA hydratase in the α-subunit catalyzes the conversion of enoyl-CoA to 3-hydroxy acyl-CoA. Next, 3-hydroxy acyl-CoA is oxidized to form 3-keto acyl-CoA by the enzyme LCHAD. The final step of β-oxidation is catalyzed by thiolase, which generates acetyl-CoA and a fatty acyl-CoA. The cycle of β-oxidation proceeds with a shorter fatty acyl-CoA for the continuous energy demand and supply of acetyl-CoA. Pediatric defects in mitochondrial fatty acid oxidation (FAO) are recessively inherited and have emerged as an important group of inborn errors of metabolism with clinical significance. Affected patients with mitochondrial FAO defects usually present in the first year of life with a Reye’s-like syndrome, cardiomyopathy, and neuro-myopathy. Death quickly ensue unless the disorder is rapidly recognized and treated.

### 1.2. Peroxisomal and Microsomal Fatty Acid Oxidation

In the event of defective mitochondrial β-oxidation due to a mutation in the β-oxidation enzymes, long chain fatty acids can be channeled to peroxisomal β-oxidation and microsomal ω-oxidation [5,6]. Unlike mitochondrial fatty acid oxidation, peroxisomal β-oxidation is not coupled with the mitochondrial electron transport chain and ATP generation. Increased peroxisomal β-oxidation would increase the production of hydrogen peroxide as a byproduct of the peroxisomal acyl-CoA oxidase, a rate-limiting enzyme and first step of peroxisomal β-oxidation. The rest of the peroxisomal β-oxidation pathway is similar to the mitochondrial β-oxidation pathway with the exception of the presence of a peroxisomal bifunctional protein, which contains an N-terminal peroxisomal enoyl-CoA hydratase and a C-terminal region that contains 3-hydroxy acyl-CoA dehydrogenase activity. Increased reactive oxygen species from the peroxisomal β-oxidation can lead to redox imbalance, mitochondrial dysfunction and cellular injury [7]. Further, accumulated fatty acids and 3-hydroxy fatty acids can also be shunted to the endoplasmic reticulum for microsomal ω-oxidation. In the liver, peroxisome proliferator-activator receptor (PPAR)-α transcriptionally regulates genes that encodes for both peroxisomal β-oxidation and microsomal ω-oxidation enzymes [6]. Enhanced microsomal ω-oxidation of fatty acids and 3-hydroxy fatty acids results in increased levels of long chain 3-hydroxy dicarboxylic acids, which then become the substrates for peroxisomal β-oxidation, leading to increased generation of reactive oxygen species as a byproduct of peroxisomal β-oxidation [1,2,8]. Increased dicarboxylic acyl-carnitines and 3-hydroxy dicarboxylic acids in plasma, and dicarboxylic acid excretion in the urine have also been reported in-patient with acute fatty liver of pregnancy (AFLP) and long-chain hydroxy acyl-CoA dehydrogenase (LCHAD) deficient children [9,10,11,12]. Further, long-chain 3-hydroxy dicarboxylic acids were shown to inhibit mitochondrial fatty acid oxidation and can act as more potent lipotoxic fatty acid intermediates compared to long chain fatty acids [13,14].

Patients with metabolic disorders of β-oxidation have toxic high levels of free fatty acids and 3-hydroxy fatty acids, both of which can be metabolized by microsomal ω-oxidation in the liver by a family of cytochrome p450 enzymes to form dicarboxylic acid [5]. Microsomal ω-oxidation of long-chain 3-hydroxy fatty acids in the human liver are predominantly catalyzed by the cytochrome p450 4F (CYP4F) gene subfamily enzyme, CYP4F11 [5]. Patients with β-oxidation defects excrete high levels of organic dicarboxylic acids in the urine and show nonketotic dicarboxylic aciduria. Several studies have shown that fibroblasts isolated from patients with medium-chain 3-hydroxy acyl-CoA dehydrogenase (MCHAD) and LCHAD deficiency demonstrate increased generation of medium chain and long chain 3-hydroxy dicarboxylic acid, respectively [9,13,14,15,16]. In summary, peroxisomal and microsomal fatty acid oxidation pathways compensate during defective mitochondrial fatty acids oxidation and result in more damage rather than protection by producing toxic fatty acid metabolites, thereby increasing the generation of reactive oxygen species leading to oxidative damage and injury.

## 2. Maternal Liver Disease Associated with Fatty Acid Oxidation Defects

Pregnancy-related diseases like hyperemesis gravidarum, preeclampsia, hemolysis, elevated liver enzymes and low platelets count (HELLP) syndrome and acute fatty liver of pregnancy were reported to be associated, to a variable degree, with the genetic defects of long chain 3-hydroxy acyl-CoA dehydrogenase (LCHAD) enzyme encoded by the *HADHA* gene in the mitochondrial β-oxidation pathway [3,17]. *HADHA* gene codes for enoyl-CoA hydratase and LCHAD, which catalyze the second and third steps in the mitochondrial β-oxidation cycle, respectively. Decreased LCHAD activity due to the mutation results in the accumulation of 3-hydroxy fatty acids. Hyperemesis gravidarum is a severe, persistent vomiting sickness that develops in the first trimester of pregnancy and has been reviewed extensively [18,19,20,21]. Hyperemesis gravidarum has been shown to be associated with the LCHAD deficiency and accumulation of 3-hydroxy fatty acids in the placenta and liver causing mild liver damage [20,22]. 

Preeclampsia is a pregnancy-related disease characterized by high blood pressure and increased urinary protein excretion that may present with signs of liver and renal damage early in the second trimester of pregnancy [23,24]. Fatty acid oxidation defects like LCHAD deficiency were reported to be associated with the development of severe preeclampsia and HELLP syndrome [25]. It has been shown in earlier studies that LCHAD mRNA, protein expression, and enzyme activity were decreased in the placenta of preeclamptic patients [26,27,28]. Also, mitochondrial long-chain fatty acid oxidation were found to be decreased in the placenta obtained from preeclamptic patients compared to controls suggesting that decreased placental fatty acid oxidation or defect in LCHAD could contribute to the pathogenesis of preeclampsia [26]. 

### 2.1. Acute Fatty Liver of Pregnancy

Acute fatty liver of pregnancy (AFLP) is an obstetric and medical emergency to the pregnant mother and unborn fetus that develops in the third trimester of pregnancy. Maternal AFLP is highly associated with carrying a fetus deficient in the LCHAD enzyme [3]. The predominant mutation reported in the *HADHA* gene is at the exon 15, 1528G>C, resulting in a loss or dramatic decrease in LCHAD activity with normal enoyl-CoA hydratase activity [3,19]. Maternal and fetal demise in AFLP are predicted to be 10% and 45%, respectively. Women with AFLP initially show common symptoms and features of liver disease during their pregnancy that rapidly progress to renal failure, coagulopathy, ascites and hepatic encephalopathy. Accurate diagnosis of AFLP requires histological evidence of hepatic microvesicular steatosis [17,29,30]. Recently, the Swansea criteria have been commonly used following the publication of criteria observed in most women with AFLP [31,32]. However, questions were raised regarding the accuracy of the Swansea criteria in diagnosing AFLP without histological evidence of hepatic microvesicular steatosis [18,33]. Patients with AFLP show a dramatic increase in the circulating biomarkers for hepatocyte and biliary injury like alanine amino transferase (ALT), aspartate amino transferase (AST), alkaline phosphatase (ALP) and γ-glutamyl transpeptidase (GGT) with increased prothrombin time [8]. 

Ibdah et al. [25] reported a strong association between fetal LCHAD deficiency and development of maternal AFLP. In a series of studies, Ibdah et al. demonstrated that women who carry LCHAD deficient fetuses with documented mutations in the *HADHA* gene develop maternal AFLP [25,34,35,36]. Treatment of LCHAD deficient children includes consumption of a diet rich in carbohydrates and medium chain fatty acids to substitute for the fatty acid demand and energy requirements, especially during fasting periods [37,38]. Medium chain fatty acids are transported to the liver through an enterohepatic portal vein for the energy demand. However, long chain fatty acids are transported as triglycerides via chylomicrons through lymphatic circulation. Further, medium chain fatty acid oxidation enzymes are soluble enzymes in the mitochondrial matrix. The mutation in the LCHAD active site results in the accumulation of 3-hydroxy fatty acids in the placenta. Since the fetal part of the placenta is identical to the genetic makeup of the fetus. The accumulated 3-hydroxy fatty acids produced in the placenta are shunted to maternal circulation, leading to the acute liver injury observed in AFLP patients [10,39]. 

### 2.2. The Incidence of AFLP and LCHAD Mutations

The incidence of AFLP is estimated to be 1 in 10,000 pregnancies in the United States. However, the incident was reported to be more frequent in some unique populations. For example, a prospective cohort study from Southwest Wales, UK reported that AFLP occurs in five out of 4377 pregnancies [40], whereas a tertiary care center in India reported that AFLP occurs in one out of 3333 pregnancies [41], and in southern India the frequency was found to be one in 6691 pregnancies [18,31,33]. Similarly, an association with mitochondrial fatty acid oxidation has been reported in patients with preeclampsia, which is a common pregnancy disorder in the United States [25,26,27,28]. At least 40% of AFLP patients have a history of preeclampsia [8,42].

The incidence of LCHAD is highly prevalent in different ethnic groups. The prevalence of LCHAD deficiency is reported to be high in Baltic Sea countries compared to other populations in the world. Recently, the LCHAD variant 1528G>C was identified to be highly prevalent in the Kashubian population of northern Poland (one carrier in 57 individuals), southern Poland (one in 107), northern Pomerania (one in 207) and isolated regions of Poland (one in 187) [43]. The 1528G>C mutation in the *HADHA* gene corresponds to the amino acid change of Glu to Gln at position 474 of the mature LCHAD domain, which resides in the active site of the enzyme. This mutation affects and reduces LCHAD catalytic enzyme activity and decreases the protein stability [44]. The LCHAD mutation is also highly prevalent in the populations of Finland (one in 240), Netherlands (one in 680), Sweden (one in 540) and Estonia (one in 173) [9,25,43,45,46,47,48,49,50,51,52]. Due to the high incidence and prevalence of LCHAD mutation, the Swedish government mandated neonatal screening for LCHAD mutation in 2012 as a routine practice to minimize the incidence of AFLP [44].

### 2.3. 3-Hydroxy Fatty Acid Accumulation

Acute fatty liver of pregnancy is highly prevalent in mothers carrying LCHAD deficient fetuses. Biochemical hallmarks of LCHAD deficiency are the accumulation of long-chain 3-hydroxy fatty acids such as 3-hydroxy lauric acid, 3-hydroxy myristic acid, 3-hydroxy palmitic acid and 3-hydroxy dicarboxylic acid in the systemic circulation and increased excretion of 3-hydroxy dicarboxylic acids in the urine [53,54,55]. Several studies support the evidence for the accumulation of long-chain 3-hydroxy fatty acids (3-HFA) in patients with LCHAD deficiency and AFLP [53,55,56,57,58,59,60,61,62,63]. Children with the LCHAD deficiency are reported to develop sudden death with hypoglycemia, cardio-respiratory failure, acute cardiac failure and insufficiency, severe neonatal cardiomyopathy, hepatic dysfunction and acute liver failure, and skeletal myopathy with rhabdomyolysis [9,62]. Further, 34% of LCHAD deficient children die between four days and 10 years after birth [9]. This multi-organ damage is believed to be due to the lipotoxicity of toxic 3-hydroxy fatty acid intermediate accumulation.

AFLP patients also accumulate saturated free fatty acids in their circulation due to the defect in the mitochondrial fatty acid oxidation [2,8]. These increased free fatty acids and 3-hydroxy fatty acids accumulate in hepatocyte, neurons, myocytes, cardiomyocytes and placental trophoblast cells. Long chain 3-hydroxy fatty acid accumulation in these cells exerts lipotoxicity, uncouples mitochondrial oxidative phosphorylation, and diminishes mitochondrial respiration.

## 3. Metabolic Phenotypes Associated with 3-Hydroxy Fatty Acid Accumulation

### 3.1. Hypoglycemia in AFLP and Hormonal Regulation

Fatty acid oxidation is an important source of energy especially for infants and children and has been reported to account for 80% of energy during initial hours of fasting [5]. Glucagon, epinephrine, norepinephrine, cortisol and growth hormones were secreted under the hypoglycemic condition to act on enhancing adipose tissue lipolysis [64]. This normal physiological function of peptide hormones enhances circulating free fatty acids levels, which then reach the liver for energy production via mitochondrial β-oxidation during hypoglycemic conditions. AFLP patients show severe hypoglycemic and hypoketotic states due to defective fatty acid β-oxidation. The levels of glucagon and stress hormones such as plasma cortisol were reported to be increased in children with LCHAD deficiency [65]. Further, we have shown increased circulating levels of long chain fatty acids like arachidonic acid, palmitic acid, myristic acid, oleic acid in patients with AFLP. AFLP patients also show increased maternal circulating long chain 3-hydroxy fatty acids such as 3-hydroxy myristic acid (3-HMA) and 3-hydroxy palmitic acid (3-HPA) due to the LCHAD defect and increased lipolysis [53,59]. Further, several case reports have shown an increased lipolysis in patients with LCHAD deficiency along with an increase in plasma dicarboxylic acid, long chain fatty acids and 3-hydroxy fatty acids after 4–6 h of fasting [9,65,66]. Recent studies have shown that lipid droplet accumulation is a protective event that packages non-esterified fatty acids as lipid droplets [67,68,69]. However, data on the hormonal regulation of hypoglycemia and peptide hormone-induced lipolysis during severe hypoglycemic conditions observed in AFLP patients are scarce and need further investigation. 

### 3.2. Mitochondrial Trifunctional Protein (MTP)-Deficient Mice Develop Intra Uterine Growth Retardation (IUGR), Neonatal Hypoglycemia, and Sudden Death

Ibdah et al. generated mice that lack both mitochondrial trifunctional protein (MTP) α and β subunits [70]. MTP homozygous knockout mice developed hepatic lipotoxicity with diffuse hepatic enlargement and lipid accumulation associated with mitochondrial swelling and damage. These MTP null mice rapidly developed cardiac and diaphragmatic lesions leading to sudden death 96 h after birth. Further, MTP null mice also showed severe intrauterine growth retardation, neonatal hypoglycemia and reported to develop hepatic microvesicular steatosis, a pathophysiological hallmark of AFLP. These MTP homozygous defective fetuses accumulate long chain fatty acids and their metabolites due to the impairment in their mitochondrial β-oxidation [70]. The cause of sudden death in fetuses with MTP null mice is likely due to the cardiac and diaphragmatic damage and possibly cardiac arrhythmias. Further, cardiac arrhythmia and sudden death are also reported in children with other fatty acid oxidation defects such as defective carnitine palmitoyl transferase 2 (CPT2) and carnitine fatty acyl-CoA translocase and MTP deficiency [20,71]. MTP^−/−^ mice were also reported to develop intrauterine growth retardation similar to the fetal phenotype observed in AFLP [70]. Thus, MTP null mice serve as a tool to study LCHAD deficiency and AFLP. 

### 3.3. MTP Heterozygous Mice Develop Hepatic Insulin Resistance

Hepatic insulin resistance was reported in MTP heterozygous mice [72]. A fifty percent reduction in the mitochondrial fatty acid oxidation and glycogen levels were reported in the hepatocytes isolated from MTP heterozygotes compared to controls. MTP heterozygous mice also showed defective insulin-induced hepatic insulin signaling pathway activation. Insulin-induced activation of insulin receptor substrate phosphorylation and their downstream targets like protein kinase B, glycogen synthase kinase 3β, forkhead family of transcription factor class O1 (FoxO1) activation were shown to be blunted in the liver from MTP heterozygous mice compared to the liver from wild-type littermates, suggesting that defective fatty acid β-oxidation causes hepatic insulin resistance [72]. This report also suggests that mitochondrial fatty acid oxidation plays an important role in hepatic insulin signaling pathways for the maintenance of normal homeostasis of liver insulin sensitivity and glycogen production. The MTP heterozygous mouse was recently used as a model for non-alcoholic fatty liver disease [73,74,75,76].

## 4. Mechanisms of 3-Hydroxy Fatty Acid-Induced Lipotoxicity

### 4.1. Placental Damage in AFLP Patients

The placenta acts as a lung, liver, and kidney for the fetus and the fetal part of the placenta is identical to the genetic make-up of the fetus. High activity of fatty acid oxidation enzymes is shown in the placenta compared to the adult liver [77,78,79,80]. Placental injury was also observed in patients with AFLP. A case report of pregnant women who developed severe AFLP showed abruptio placentae (placental abruption), premature delivery and fetal demise [81]. Maternal floor infarction of the placenta was also reported in a mother who gave birth to an LCHAD deficient child [82]. Further, placenta from AFLP patients were reported to have abnormalities and increased oil red O staining, suggesting an increase in placental lipid droplet accumulation and lipotoxicity [83].

In AFLP, a fetus homozygous for an LCHAD mutation will have defective placental metabolism of long chain fatty acids resulting in the accumulation of 3-hydroxy fatty acids and long chain fatty acids. Accumulated 3-hydroxy fatty acids and other fatty acids enter the mother’s circulation and affect the maternal liver, resulting in AFLP. Further, LCHAD deficient children have been known to display mitochondrial damage as evidenced by increased mitochondrial swelling, and irregular mitochondrial cristae in the skeletal muscle [55,57,60,61,62,63,82,84,85,86]. The cytotoxic 3-hydroxy fatty acids are also known to inhibit mitochondrial processes like β-oxidation and oxidative phosphorylation enzymes resulting in decreased ATP production and increased reactive oxygen species; thereby resulting in mitochondrial damage [55,62,87]. Although the strong association between maternal AFLP and fetal LCHAD deficiency is well-documented, there are few case reports that suggest an association with other fetal fatty acid oxidation disorders. For instance, a published report linked maternal AFLP with pediatric carnitine palmitoyl transferase I deficiency [88]. A recent report also showed that AFLP developed in a mother carrying a fetus with *HADHB* homozygous mutation [89]. The possibility that AFLP can be associated with fatty acid oxidation defects other than LCHAD deficiency is intriguing and warrants further investigation to understand the underlying molecular mechanisms. 

### 4.2. Subcellular Damage and Oxidative Injury

Subcellular damage and oxidative injury was evident in animal models of microvesicular steatosis. We have shown that inhibition of mitochondrial β-oxidation using near-lethal doses of valproate developed hepatic microvesicular steatosis and oxidative stress in the liver. Hepatic mitochondrial membrane damage and dysfunction were also evident in this rat model of microvesicular steatosis [8,90]. Further, placental mitochondria isolated from patients with AFLP showed decreased respiration, altered mitochondrial calcium homeostasis, increased superoxide generation and increased mitochondrial swelling, suggesting a placental mitochondrial dysfunction compared to controls. We have also shown a dramatic increase in the levels of oxidative injury biomarkers in placental mitochondria, peroxisomes, and microsomes in patients with AFLP compared to controls [1,2]. Further, circulating oxidative and nitrosative stress markers were increased in the maternal circulation of AFLP patients, suggesting that reactive nitrogen species act together with reactive oxygen species to damage cells. Concomitantly, circulating levels of antioxidants like tocopherols and retinol were dramatically decreased in patients with AFLP compared to controls suggesting an oxidative and nitrosative stress in the maternal systemic circulating in patients with AFLP [8]. The simultaneous presence of reactive oxygen species and nitric oxide could lead to the formation of peroxynitrite, a highly damaging oxidant.

Interestingly, a recent proteomic analysis revealed that the expression of HADHA protein was dramatically decreased in mitochondria isolated from rat livers with nonalcoholic steatohepatitis [91]. Further, HADHA protein expression was also found to be downregulated in the left ventricle of spontaneously hypertensive rats. Decreased HADHA protein expression was evident from 1–20 weeks old spontaneously hypertensive rats [92]. However, the mRNA levels were observed to be unaffected suggesting post-transcriptional mechanisms. Oxidative stress and mitochondrial dysfunction were evident in the development of non-alcoholic steatohepatitis [93] and in spontaneous hypertensive animals [94]. Together, these data suggest that oxidative stress can affect the stability of HADHA proteins in the mitochondrion. Further studies are required to validate this hypothesis on HADHA protein stabilization and its activity during cellular oxidative stress environments. 

### 4.3. 3-Hydroxy Fatty Acid-Induced Hepatocyte Lipoapoptosis

**Figure 2:**
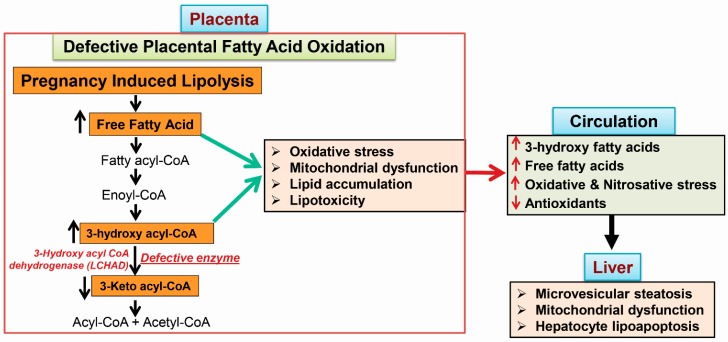
Schematic representation of the sequence of events that happen during acute fatty liver of pregnancy (AFLP). Fetal long chain 3-hydroxy acyl-CoA dehydrogenase (LCHAD) deficiency results in accumulation of 3-hydroxy fatty acids in the placenta, since the fetal part of placenta is identical to the genetic makeup of the fetus. Increased accumulation of placental free fatty acids and 3-hydroxy fatty acyl-CoA cause oxidative stress, mitochondrial dysfunction and placental lipotoxicity. Further, lipolysis induced in the third trimester of pregnancy would also trigger the accumulation of fatty acid intermediates, which are shunted from the placenta to the maternal circulation, where they can promote oxidative and nitrosative stress. These fatty acid intermediates reach the maternal liver resulting in microvesicular steatosis, hepatic mitochondrial dysfunction and hepatocyte lipoapoptosis.

We have shown that long-chain fatty acids like palmitic acid and arachidonic acid levels were dramatically elevated in the systemic circulation of patients with AFLP. The lipotoxic role of saturated free fatty acid like palmitate was reported to induce caspase-dependent hepatocyte and cholangiocyte lipoapoptosis [68,95,96,97,98]. Recently, we have also demonstrated that the signaling mechanism of palmitate-induced cholangiocyte lipoapoptosis is via the activation of mitogen-activated protein kinase (MAPK) and forkhead family of transcription factor class O3 (FoxO3) and its downstream targets like p53-upregulated modulator of apoptosis (PUMA) protein and pro-apoptotic microRNA 34a [68,99,100]. Further, we reported that the exposure of arachidonic acid to the hepatocyte similar to the concentration observed in AFLP patients showed an increased lipid droplet accumulation, mitochondrial reactive oxygen species and caspase 3 activation leading to hepatocyte lipoapoptosis. These results suggest that AFLP-related long-chain fatty acid accumulation in the maternal systemic circulation can induce hepatocyte lipoapoptosis in patients with AFLP [2,8]. Increased long chain 3-hydroxy fatty acid in patients with AFLP can also induce mitochondrial dysfunction and hepatocyte lipoapoptosis. Our unpublished preliminary data show that the treatment of long chain 3-hydroxy fatty acids (3-HFAs) such as 3-hydroxy myristic acid (3-HMA) and 3-hydroxy palmitic acid (3-HPA) to cultured hepatocytes results in caspase-dependent hepatocyte lipoapoptosis. However, interestingly, short chain 3-hydroxy octanoic acid (3-HOA) did not induce hepatocyte lipoapoptosis. Similar to mitochondrial fatty acid oxidation defects, mice lacking peroxisomal fatty acyl-CoA oxidase also show microvesicular steatosis, hepatocyte apoptosis and liver injury [101]. A schematic presentation of the sequence of events that occur in-patient with AFLP is shown in Figure 2. Experiments to elucidate the mechanism of 3-HFA-induced hepatocyte lipoapoptosis are currently underway in our laboratory. 

## 5. 3-HFA and Pediatric Complications Unique to LCHAD Deficiency

**Figure 3:**
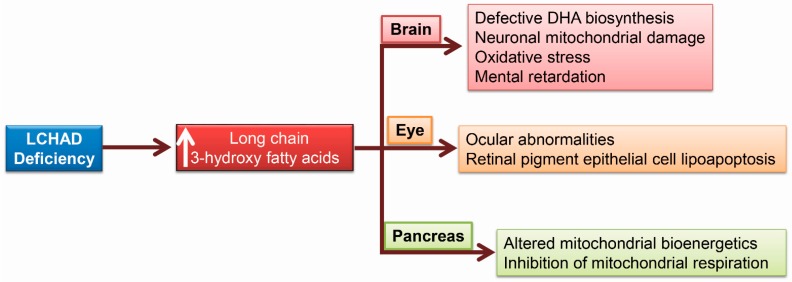
Other complications due to 3-hydroxy fatty acid (3-HFA) accumulation. LCHAD deficiency in children results in docosahexenoic acid (DHA) deficiency and 3-HFA accumulation induces neuronal mitochondrial and oxidative damage in the brain. LCHAD deficiency was also associated with ocular abnormalities and retinal pigment epithelial cell lipoapoptosis. In the pancreas, 3-HFA alters mitochondrial bioenergetics in the islet β-cells.

LCHAD deficient children accumulate long chain 3-hydroxy fatty acids that cause brain damage and ocular abnormalities as shown in Figure 3.

### 5.1. Brain Damage Due to 3-Hydroxy Fatty Acids

LCHAD deficiency results in the development of brain damage and injury due to the accumulation of 3-HFAs. LCHAD-deficient patients were also shown to have defective brain docosahexaenoic acid (DHA) biosynthesis due to the defect in mitochondrial β-oxidation, since β-oxidation in the brain is critical for the final step in DHA biosynthesis [11,102]. Studies have shown that patients with LCHAD deficiency were also more likely to develop seizures, mental retardation, retinopathy and neuropathy, possibly due to the defect in DHA biosynthesis [43,48,51,53,54,103]. LCHAD deficient children also show exercise intolerance, early development disability, mental retardation, microcephaly, skeletal myopathy, cardiomyopathy, encephalopathy in addition to hepatomegaly with microvesicular steatosis. These pathological abnormalities are due to the accumulation of toxic 3-HFAs and 3-hydroxy dicarboxylic acids. Increased 3-HFA accumulation in the brain has been postulated to induce oxidative damage to the neurons leading to the development of neurological symptoms observed in patients with LCHAD deficiency [51,53,54,55]. Further, rat cerebral cortex incubated with 3-HFA showed increased oxidative damage, as evident by an increase in biomarkers of oxidative injury and decreased antioxidant status [55]. These data support the hypothesis that 3-HFA exerts lipotoxicity to the brain and induces oxidative stress and can induce neuronal cell lipoapoptosis in LCHAD deficiency.

### 5.2. Ocular Abnormalities in Patients with LCHAD Deficiency

Ocular damage and poor vision is a unique defect observed in patients with LCHAD deficiency that is not common to other inborn errors of fatty acid oxidation defects. Recent studies have shown that LCHAD-deficient individuals develop retinal dysfunction and chorioretinopathy leading to subnormal vision function [48,102,104,105,106]. The ocular damage is typically accompanied by retinal pigmentation, peripheral neuropathy and cognitive deficiency [48,103]. The mechanism of chorioretinopathy and ocular abnormalities remains poorly understood. However, the accumulation of 3-hydroxy fatty acids and 3-hydroxy dicarboxylic-acid-induced lipotoxicity could contribute to these pathophysiological changes [48]. A recent study generated an induced-pluripotent stem cells (iPSC) from fibroblasts obtained from patients with the LCHAD homozygous mutation (1528G>C). The iPSCs were differentiated into retinal pigment epithelial (RPE) cells, in vitro. RPE cells obtained from patients with LCHAD homozygous mutation showed smaller size, irregular shape, decreased pigmentation, decreased melanosomes and enhanced melanolysosmes compared to control cells. These results suggest that depigmentation and melanosome degradation is involved in the development of retinal pigment retinopathy associated with LCHAD mutations. RPE cells derived from LCHAD mutant patients also showed a dramatic decrease in the expression of Na^+^/K^+^ ATPase and tight junction protein, zona occludens-1 (ZO-1), compared to control-derived cells. Also, lipid droplet accumulation with increased triglycerides levels was evident in RPE cells derived from LCHAD-deficient individuals. Further, increased apoptosis were reported in RPE cells derived from patients with a LCHAD mutation [107]. We hypothesize that increased levels of toxic 3-hydroxy fatty acid intermediates of mitochondrial β-oxidation could be involved in the development of the ocular injury, by inducing lipoapoptosis to the retinal pigment epithelial cells during LCHAD deficiency. However, further studies are needed to elucidate the molecular mechanism of 3-hydroxy fatty acid-induced RPE cell lipoapoptosis secondary to an LCHAD defect. 

### 5.3. 3-Hydroxy Fatty Acids Alter Pancreatic Islet β-Cell Bioenergetics

AFLP is also associated with the development of pancreatitis [108,109,110,111,112,113,114]. The role of 3-hydroxy fatty acids in relation to the development of pancreatitis remains unexplored. However, a recent study showed that glucolipotoxicity, i.e., hyperglycemia and elevated blood lipids results in the accumulation of 3-hydroxy fatty acid and exerts its lipotoxic function by impairing the pancreatic β-cell mitochondrial bioenergetics due to the uncoupling and inhibition of mitochondrial respiration [115]. Glucolipotoxicity to human and mouse primary islets cells results in the increased expression of carnitine palmitoyl transferase 1A, long-chain acyl-CoA dehydrogenase, and peroxisome proliferator-activated receptor-γ coactivator-1α (PGC1α), a transcription factor that acts on enhancing fatty acid oxidation enzymes. Further, glucolipotoxicity results in decreased levels of acetylcarnitine, suggesting an incomplete mitochondrial β-oxidation [115]. However, this study did not show whether the incomplete β-oxidation was due to the decreased LCHAD activity or decreased *HADHA* gene expression. Further studies are warranted to elucidate the exact mechanism of 3-hydroxy fatty-acid-induced pancreatic islet β-cell impairment during glucolipotoxicity and the development of pancreatitis during AFLP.

## 6. Conclusions

In conclusion, 3-hydroxy fatty acids and long chain fatty acids accumulate in patients with acute fatty liver of pregnancy (AFLP) from the fetal placenta. These toxic 3-hydroxy fatty acid intermediates shunted from the placenta to the maternal circulation induce catastrophic acute maternal liver injury. Further, LCHAD-deficient patients also accumulate 3-hydroxy fatty acids and develop mental retardation, developmental disabilities, ocular abnormalities and sudden infant death. The mechanism of 3-HFA-induced lipotoxicity is underway in our laboratory. 

## Abbreviations

AFLP	acute fatty liver of pregnancy
ALT	alanine amino transferase
AST	aspartate amino transferase
ALP	alkaline phosphatase
CPT2	carnitine palmitoyl transferase 2
DHA	docosa hexaenoic acid
FoxO	forkhead family of transcription factor class O
GGT	γ-glutamyl transpeptidase
HELLP	hemolysis elevated liver enzymes with low platelet count
iPSC	induced-pluripotent stem cells
LCHAD	long chain hydroxy acyl-CoA dehydrogenease
MAPK	mitogen activated protein kinases
MTP	mitochondiral trifunctional protien
MCHAD	medium chain hydroxy acyl-CoA dehydrognease
PGC1α	peroxisome proliferator-activated receptor-γ coactivator-1α
PUMA	p35-upregulated modulator of apoptosis
RPE	retinal pigment epithelial cells
TCA cycle	tricarboxylic acid cycle
ZO-1	zona occludens-1
3-HFA	3-hydroxy fatty acid
3-HMA	3-hydroxy myristic acid
3-HOA	3-hydroxy octanoic acid
3-HPA	3-hydroxy palmitic acid
